# Change in Practice over Four Decades in the Management of Graves' Disease in Scotland

**DOI:** 10.1155/2016/9697849

**Published:** 2016-05-30

**Authors:** D. M. Smith, S. Dutta, F. Ahmed, M. A. Thaha

**Affiliations:** ^1^Department of Surgery, Ninewells Hospital & Medical School, University of Dundee, Dundee DD1 9SY, UK; ^2^University Department of Surgery, Faculty of Medicine, University of Glasgow, Royal Infirmary, Glasgow G31 2ER, UK; ^3^School of Medicine, Guy's King's & St. Thomas' Hospitals, King's College London, London SE1 1UL, UK; ^4^Academic Surgical Unit, Blizard Institute, National Centre for Bowel Research & Surgical Innovation, Barts and The London School of Medicine & Dentistry, Queen Mary University of London, London E1 1BB, UK

## Abstract

There is continuing debate on the optimal treatment for Grave's thyrotoxicosis with a resultant variation in clinical practice. The present study aimed to ascertain changes in practice in the treatment of Grave's thyrotoxicosis in Tayside, Scotland, over the past four decades.* Methods*. The “Scottish automated follow-up register” (SAFUR) was queried to identify all patients treated for Grave's thyrotoxicosis from 1968 to 2007 inclusive. Patients were divided into 4 groups (Groups A to D) according to the decades. Demographic profile, treatment modalities, radioactive iodine (RAI) dose, and recurrence rates were studied and outcomes were compared by *χ* ^2^ test and ANOVA using SPSS v15.0. A *p* value of < 0.05 was considered significant.* Results*. Altogether, 3737 patients were diagnosed with Grave's thyrotoxicosis over the 4 decades. Use of RAI has increased from 43.1% in Group A to 68% in Group D (*p* < 0.001). The dose of RAI has increased (*p* < 0.001) and there has been a reduction in recurrence rate with higher dose of RAI. Surgical intervention rates decreased from 55.3% to 12.3% (*p* < 0.001) over time.* Conclusions*. Analysis of a large dataset of patients with Grave's thyrotoxicosis suggests increasing use of RAI as the preferred first line of treatment. Furthermore, using a single higher dose of RAI and adoption of total thyroidectomy have decreased recurrence rates.

## 1. Introduction

Graves' thyrotoxicosis, an autoimmune thyroid disorder affecting about 0.5% of the population, is the most common cause of diffuse toxic goiter [1]. It accounts for 60 to 80% of patients with hyperthyroidism and shows a female preponderance with a female : male ratio of 10 : 1 [2]. The three distinct treatment modalities (antithyroid drugs (ATDs), radioactive iodine (^131^I) therapy (RAI), or surgery (total, subtotal, or partial thyroidectomy)) commonly used in the treatment of Graves' thyrotoxicosis are used either singularly or in variable combinations depending on circumstances including disease characteristics and response to initial treatment. The choice of optimal initial therapy for a patient with newly diagnosed Graves' thyrotoxicosis is influenced by multiple factors and this may include patient/disease related factors, physician related factors, and system related factors such as cost [3, 4]. Furthermore, cultural and geographical influences are also thought to play a role in the choice of initial treatment offered to and accepted by patients [5].

The prevailing differences in treatment preferences for Graves' thyrotoxicosis have been highlighted by surveys conducted in America, Europe, and Japan [5]. In addition, studies have reported a similar early outcome of the disease, irrespective of the initial treatment modality used. However, there was a clear difference in the relapse rate with different treatment, surgery producing the lowest relapse rates and antithyroid drugs producing the highest [6]. The linear association between RAI dosage and remission rates has been investigated and recorded previously [7]. Furthermore, there is currently no consensus on the best surgical option to treat Grave's thyrotoxicosis.

Given the continuing debate on the optimal index therapy for Graves' thyrotoxicosis, in the current study, we investigated the time trends in the choice of initial therapy used for a large cohort of patients with newly diagnosed Graves' thyrotoxicosis over a period of four decades. We also sought to establish the remission rates with different treatment modalities including variable doses of RAI and different types of surgery that was used over the forty-year study period.

## 2. Methods

“Scottish automated follow-up register” (SAFUR) is a computer-based, shared-care (between primary care and hospital clinic) facility for automated follow-up of patients diagnosed with thyroid disorders including hyperthyroidism in Scotland [8]. SAFUR has been available for clinical use since 1968 and currently has more than 20,000 patients registered on the database. In Tayside, Scotland, over the past two decades, a well-defined clinical pathway exists for the care of patients with endocrine disorders including Grave's thyrotoxicosis. All patients with thyroid disorders referred by the general practitioner to the hospital are first seen by a medical endocrinologist who initiates the necessary investigations and appropriate treatment from a hospital based thyroid clinic. Patients are referred to an endocrine surgeon by the medical endocrinologist for consideration of operative treatment based on their clinical response. Postsurgical follow-up in the initial one year after surgery is by the surgical endocrine team and beyond one year further long-term follow-up will be led by the medical endocrine team. Patients are rereferred to the surgeons for any surgical related problems. At initial diagnosis patients are registered into the SAFUR database, a nation-wide follow-up registry which has an automated function for periodic call back of patients for surveillance blood checks at predetermined times. The test results are reviewed and modifications to treatment based on the test results are made by the hospital based SAFUR medical endocrinology team.

From the SAFUR database, we identified all patients who were newly diagnosed with Grave's thyrotoxicosis in Tayside health board region, Scotland (estimated 2010 midyear population of 402,641) during the period 1968–2007 inclusive. Diagnosis of Graves' disease was based on clinical findings and elevated free T4 with or without an isotope scan. Patients with toxic adenoma or toxic multinodular goiter causing thyrotoxicosis were excluded. For each patient the demographic data (age, gender) and clinical data including treatment data (index treatment, second treatment, use of RAI, dose of RAI used, use of surgery, and surgical technique employed) were collated. Age at diagnosis was calculated from the date of receiving first treatment. We determined the remission and recurrence rates based on the serum TSH levels recorded in the SAFUR database. For the study purposes, any normal or increased TSH level after index treatment was considered as remission. Patients who received a second treatment and suppressed THS level after a period of euthyroidism or hypothyroidism were considered to have a clinically significant recurrence.

Patients were divided into four groups according to the decade studied (Group A = 1968–1977; Group B = 1978–1987; Group C = 1988–1997; and Group D = 1998–2007) and outcome measures were compared between the four groups to identify any time trends. RAI dose was grouped in five different levels (<185 MBq, 185–369 MBq, 370–554 MBq, 555–740 MBq, and >740 MBq). Age is presented as mean ± SD. Recurrence rates are presented as percentages. The data was analysed using SPSS software (Statistical Package for the Social Sciences version 15.0, SPSS Inc., Chicago, IL, USA) and comparisons were made using *χ* ^2^ test and ANOVA. A *p* value of < 0.05 was considered significant.

## 3. Results

### 3.1. Baseline Characteristics

Between 1968 and 2007, 3737 patients were diagnosed with Graves' disease in Tayside health board region of Scotland. The number of newly registered patients with a diagnosis of Graves' disease increased with each decade of the study period: Group A representing the first decade from 1968 to 1977 (*n* = 436); Group B from 1978 to 1987 (*n* = 755); Group C from 1988 to 1997 (*n* = 1185); and Group D representing the last decade from 1998 to 2007 (*n* = 1361) (Table 1). There was a female predominance (*n* = 3155, 84.4%) in the whole study group with an overall female-to-male ratio of 5.4 : 1 (Table 1). This preponderance of female patients was stable over the forty-year study period. However, a statistically significant increase in the proportion of male patients diagnosed with Graves' disease was noticed through the four decades (12.6% versus 16.8%: Group A versus Group D; *p* = 0.035) studied (Table 1).

**Figure 1:**
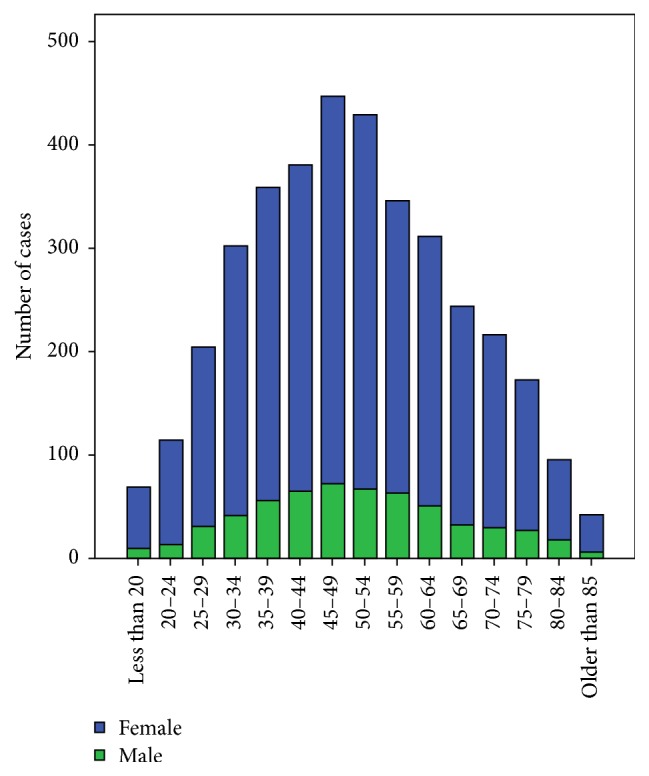
Age distribution of patients with Graves' disease.

The mean age at diagnosis for the whole study group was 50 ± 16 years (Table 1). The overall mean age of patients with Graves' disease increased through the four decades and this was statistically significant (43 ± 12 versus 53 ± 17: Group A versus Group D; *p* < 0.001) (Table 1). This increase in mean age was true when analysed by gender, with both males and females showing an increase through the four decades.  Figure 1 shows the age distribution for both genders and no differences in the mean age between genders were noticed over the four decades.

### 3.2. Index Therapy

The choice of index therapy used during the four decades is shown in Table 1. Overall, antithyroid drugs remained the least commonly used agent for initiating treatment after diagnosis through the four decades. However, its use as the first-line agent almost doubled in the last decade. In the first decade nearly half of the patients (48.2%) received surgery as the first-line treatment. However, a progressive decline in the use of surgery is recorded through the decades with only 11.6% patients undergoing surgery as principal treatment for Graves' disease in the last decade. This is in contrast to the gradual increase in the use of RAI (36% versus 67.3%: Group A versus Group D) over the four decades. The combined use of surgery with postoperative RAI ablation also saw a decline (7.1% versus 0.7%: Group A versus Group D) through the four decades (Table 1). This shift from surgical to medical treatment was found to be statistically significant (*p* < 0.001).

### 3.3. Surgery as Index Treatment

**Figure 2:**
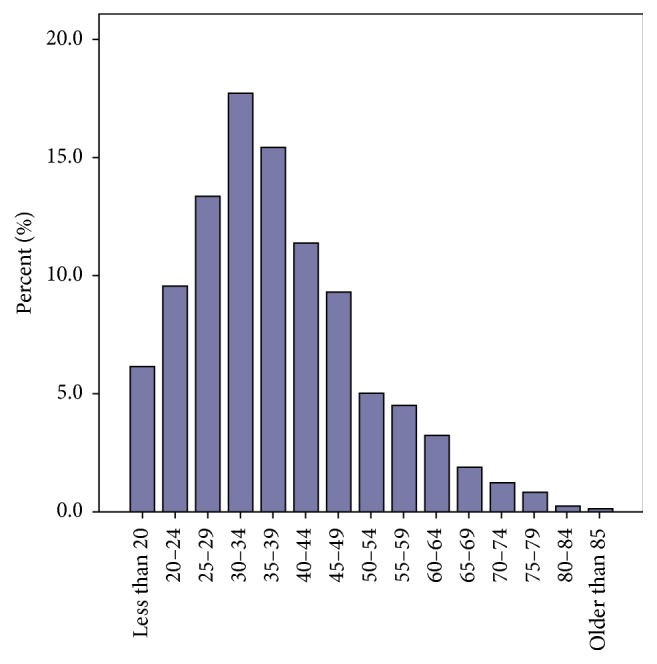
Age distribution of patients who underwent surgery.

The mean age of patients who had surgical treatment as index therapy was 37.5 ± 13.5 years.  Figure 2 shows the age distribution of patients receiving surgery. The operation of choice during the first two decades (Groups A and B) was subtotal thyroidectomy. The surgical practice changed in 1992 to near total or total thyroidectomy. Consequently, Groups C and D patients had either near total or total thyroidectomy. There was a downward trend in the recurrence rates after surgical treatment (11.4% versus 3%: Group A versus Group D) over the decades and this trend is likely to reflect the change in surgical practice from subtotal thyroidectomy to total thyroidectomy (Table 2). The mean time from surgical treatment to recurrence was 127 ± 91 months (range: 4–338 months). On the other hand, the rate of postoperative hypothyroidism increased from 75% (Groups A and B) to 96.4% (Groups C and D) reflecting the use of total thyroidectomy as the preferred surgical technique.

### 3.4. RAI as Index Treatment

**Figure 3:**
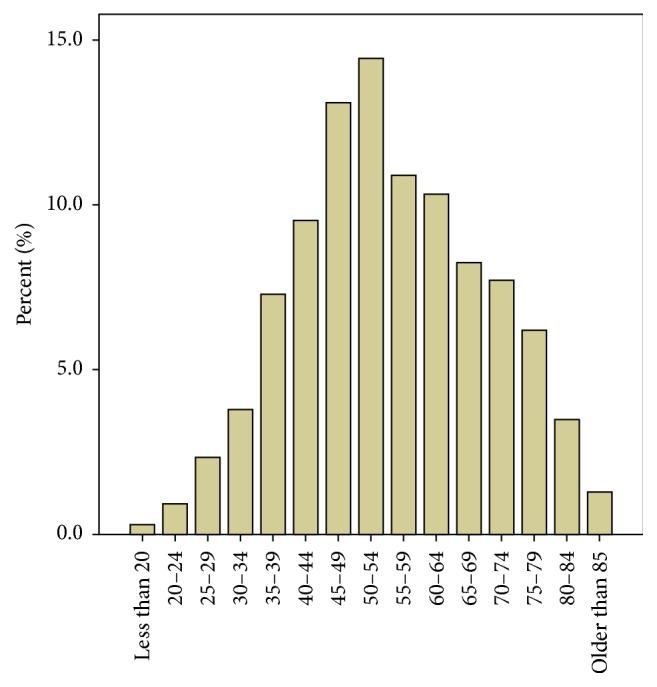
Age distribution of patients who had RAI treatment.

**Figure 4:**
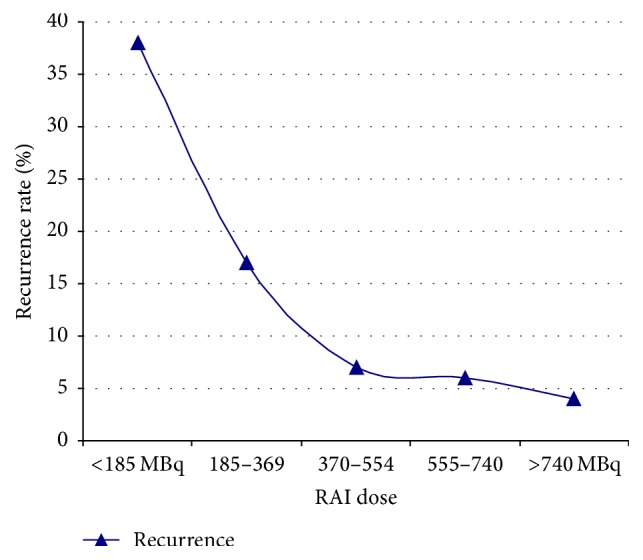
Recurrence rate in relation to RAI dose.

Patients who received treatment with RAI had a mean age of 55 ± 14.5 years.  Figure 3 demonstrates the age distribution of patients receiving RAI as primary treatment. The use of RAI as index therapy increased through the decades from 36% in the first decade (group A) to around 70% in the last two decades (Group C and Group D) (Table 1) of the study period. Altogether 2358 patients had RAI but information on dose of RAI used was available for 2237 (94.9%) patients only. There was a trend towards usage of higher doses of RAI over the time (*p* < 0.001). This was associated with a concurrent reduction in recurrence rates following RAI ablation therapy (*p* < 0.001) (Figure 4). The complete remission rate following RAI treatment was 92.8% with 7.2% patients having recurrence following RAI treatment. This recurrence rate is comparable to the overall recurrence rate of 6.3% following surgical treatment in the study. Only 8.6% of patients, however, treated with ATD showed a complete remission.

A second dose of RAI was administered to 158 patients with a mean dose of 602.8 ± 222.5 MBq. Thyroxine replacement was required in 87.2% of patients (*n* = 2358) following RAI therapy, a figure that was comparable to the rates of postoperative hypothyroidism seen in patients treated surgically. The rate of posttreatment hypothyroidism was independent of the RAI dose at long-term follow-up (Table 3).

## 4. Discussion

Treatment approaches to Grave's disease differ, but ultimately they are dependent on age, existing comorbidities, and preference of patients and clinicians. Although most clinicians would recommend antithyroid drugs (ATDs) as the first-line therapy [1], variations in practice have been recorded between Europe, Asia, and America [9]. Whilst, in America, ATDs are used as an adjunct prior to index treatment with RAI or surgery [5], in Europe and Asia ATDs are primarily employed as the initial therapy for newly diagnosed cases with RAI or surgery was offered as second-line treatment for those patients who relapse on ATD. Surgical treatment may be the preferred option for many patients as it provides immediate symptomatic relief, does not require frequent monitoring of thyroid function since the inevitable hypothyroidism is treated with hormone replacement therapy, and interferes less with patients' complex lifestyles [4]. Furthermore, recent cost-effectiveness studies have demonstrated that thyroidectomy might be more cost-effective than RAI or ATDs, without compromising quality of life [10–12].

In view of the controversies in the management of GD, this study, one of the largest to date, attempted to summarise, compare, and contrast the management of patients with Graves' thyrotoxicosis in a health authority in Scotland over a period of four decades.

Antithyroid medication was consistently the least popular definitive treatment modality probably due to its associated high recurrence rate. The reported remission rate with ATDs is around 40%–50% [9] but, in our long-term series, it was only 8.6%. Furthermore, lifelong ATD therapy necessitates frequent thyroid function monitoring and is associated with life-threatening side effects such as agranulocytosis and hepatotoxicity [13]. Surprisingly, the use of ATDs almost doubled in the last decade (Table 1), which is difficult to explain but may be related to the increasing age of patients diagnosed with Graves' disease. Older patients may be more likely to opt for conservative management and younger patients for surgery since the mean age of patients who had surgery as index therapy was 37.5 years. This is supported by other studies who reported that patient preference was the most common indication for thyroidectomy [4].

Another interesting trend identified regarding the choice of therapy was the significant decline in the use of thyroidectomy alone or in combination with RAI and the impressive increase in the use of RAI. This is likely to be the result of the increasing willingness to utilize RAI because of its safety, the ease and convenience of administration, its lower cost, and increasing acceptance by patients [3, 5, 9, 14]. Furthermore, RAI has similar remission and posttreatment hypothyroidism rates to thyroidectomy. Despite RAI becoming the first line of definitive therapy in Graves' disease, there is lack of consensus regarding optimal treatment dose [1]. Treatment with adjusted RAI dose has failed to prove superiority over fixed standard dose regimen [15, 16]. In our study, remission rate with a dose of 370–740 MBq is higher (~93%) than that of other published studies [17, 18]. However, an increased rate of remission (96.2%) has been achieved with a dose of >740 MBq (741–1110 MBq) and is similar to that reported in another study [7]. Furthermore, the rate of hypothyroidism is fairly common in all groups and not directly related to the RAI dose.

Despite its large sample size obtained from a national registry our study has the following limitations. The Scottish national statistics reported an increase in the midterm population of the Tayside health board region (e.g., midterm population; 1981 versus 2010; 397,055 versus 402,641) during the four decades of study period. However, as the geographical region covered by the Tayside health board underwent reorganisation during this period, we were unable to calculate the true changes in population-based incidence of Grave's disease for the region. Despite its being a prospective registry not all data was available for the use of RAI and particularly for the RAI dosage. The final analysis reported in the study used the available dataset [19]. Furthermore, the scope of the study was limited to a single health board region with all the comparisons made being “within study” comparisons to ascertain changes with time through the four decades studied. No attempt was made to compare the health board data with the national data, as this was not available for the study group at the time of the study.

In conclusion, the treatment of Graves' disease in a Scottish Health Authority has evolved over the last 40 years favouring the early use of RAI in almost 70% of patients. This is compatible with common practice in the United States where the vast majority of patients are treated with RAI with or without ATDs. A higher dose of radiation (>740 MBq) is recommended to prevent recurrence and achieve remission. Further development of clinical pathways should be informed by the outcomes of this longitudinal observational study.

## Figures and Tables

**Table 1:** Demographic characteristics and index therapy according to decades.

	Whole study 1968–2007	Group A 1968–1977	Group B 1978–1987	Group C 1988–1997	Group D 1998–2007
Total number of GT patients (%)	3737 (100%)	436 (11.7%)	755 (20.2%)	1185 (31.7%)	1361 (36.4%)
*Gender distribution*					
Females (%)	3155 (84.4%)	381 (87.4%)	642 (85%)	1000 (84.4%)	1132 (83.2%)
Males (%)	582 (15.6%)	55 (12.6%)	113 (15%)	185 (15.6%)	229 (16.8%)
Female : male	5.4 : 1	6.9 : 1	5.7 : 1	5.4 : 1	4.9 : 1
Overall age Mean ± std dev	50 ± 16	43 ± 12	47 ± 15	51 ± 16	53 ± 17
Age of females Mean ± std dev	50 ± 16	43 ± 12	47 ± 15	51 ± 16	53 ± 17
Age of males Mean ± std dev	51 ± 16	41 ± 13	49 ± 14	50 ± 17	54 ± 16
*Index treatment used*					
Antithyroid drugs	320 (8.6%)	27 (6.2%)	47 (6.2%)	76 (6.4%)	170 (12.5%)
Radioactive iodine	2274 (60.1%)	157 (36%)	371 (49.1%)	830 (70%)	916 (67.3%)
Surgery	873 (23.4%)	210 (48.2%)	293 (38.8%)	212 (17.9%)	158 (11.6%)
Surgery + RAI	87 (2.3%)	31 (7.1%)	28 (3.7%)	18 (1.5%)	10 (0.7%)

GT: Graves' thyrotoxicosis; std dev: standard deviation; RAI: radioactive iodine.

**Table 2:** Outcomes after thyroidectomy according to decade.

	Total number of patients (%)	Remission rate (%)	Recurrence rate (%)
Group A (1968–1977)	241 (55%)	214 (89%)	11.4%
Group B (1978–1987)	321 (43%)	295 (92%)	7.3%
Group C (1988–1997)	229 (19%)	217 (95%)	3.2%
Group D (1998–2007)	167 (12%)	167 (97%)	3%

**Table 3:** Outcome after different RAI dose.

RAI dose	Remission *n* (%)	Recurrence *n* (%)	Hypothyroidism *n* (%)	Total
<185 MBq	5 (62.5)	3 (37.5)	5 (62.5)	8
185–369 MBq	244 (82.7)	51 (17.3)	265 (89.8)	295
370–554 MBq	1046 (93.5)	73 (6.5)	989 (88.4)	1119
555–740 MBq	74 (93.7)	5 (6.3)	70 (88.6)	79
>740 MBq	708 (96.2)	28 (3.8)	617 (83.8)	736
*Total*	2077 (92.8)	160 (7.2)	1946 (86.9)	2237
